# Improvement of Breast Cancer Detection Using Dual-Layer Spectral CT

**DOI:** 10.3390/diagnostics14141560

**Published:** 2024-07-19

**Authors:** Felix Christian Hasse, Athanasios Giannakis, Eckhard Wehrse, Wolfram Stiller, Markus Wallwiener, Hans-Ulrich Kauczor, Tim F. Weber, Jörg Heil, Theresa Mokry

**Affiliations:** 1 Diagnostic and Interventional Radiology (DIR), Heidelberg University Hospital, Im Neuenheimer Feld 420, 69120 Heidelberg, Germanytim.weber@med.uni-heidelberg.de (T.F.W.); 2 Division of Radiology, German Cancer Research Center (DKFZ), Im Neuenheimer Feld 280, 69120 Heidelberg, Germany; 3 Department of Obstetrics and Gynecology, Heidelberg University Hospital, Im Neuenheimer Feld 440, 69120 Heidelberg, Germany; 4 Heidelberg Breast Centre St. Elisabeth Clinic, Max-Reger-Straße 5, 69121 Heidelberg, Germany

**Keywords:** thorax, breast neoplasms, multidetector computed tomography

## Abstract

This study aimed to investigate the diagnostic performance of breast mass detection on monoenergetic image data at 40 keV (MonoE40) and on iodine maps (IM) compared with conventional image data (CI). In this prospective single-center case-control study, 50 breast cancer patients were examined using contrast-enhanced dual-layer spectral CT. For qualitative and quantitative comparison of MonoE40 and IM with CI image data, four blinded, independent readers assessed 300 randomized single slices (two slices for each imaging type per case) with or without cancerous lesions for the presence of a breast mass. Detection sensitivity and specificity were calculated and readers rated their subjective diagnostic certainty. For statistical analysis of sensitivity and specificity, a paired *t*-test and ANOVA were used (significance level *p* = 0.05). A total of 50 female patients (median age 51 years, range 28–83 years) participated. IM had the highest overall scores in sensitivity and specificity for breast cancer detection, with 0.97 ± 0.06 and 0.95 ± 0.07, respectively, compared with 0.90 ± 0.04 and 0.92 ± 0.06 in CI. MonoE40 yielded a sensitivity of 0.96 ± 0.02 and specificity of 0.94 ± 0.08. All differences in sensitivity and specificity between MonoE or IM and CI were statistically significant (*p* < 0.001). The superiority of IM sensitivity and specificity was most pronounced in patients with dense breasts. Spectral CT improved the detection of breast cancer with higher sensitivity and specificity compared to conventional image data in our study.

## 1. Introduction

Although breast cancer has a high incidence, the positive predictive value of suspicious findings in the breast on CT image data is low [1]. At the same time, primary staging of breast cancer is regularly performed using CT, focusing on locoregional lymph nodes and distant metastases. The primary tumor itself is often hard to evaluate due to poor delineation from the surrounding glandular tissue when using conventional CT. Standard assessment of the local extent of the primary tumor is performed by clinical examination, mammography/tomosynthesis, sonography, and, if necessary, magnetic resonance imaging (MRI) of the breast [2]. Increased breast density is not only associated with an increased risk of breast cancer, but also complicates diagnosis in mammography [3,4].

In contrast to conventional CT systems, dual-layer detector CT features two independent detector layers, enabling simultaneous measurement of two complete sets of CT projection data (i.e., at higher and lower photon energy, respectively), and thus allowing spectral analysis without increasing the radiation exposure or lengthening the examination time [5,6]. Radiation exposure can even be reduced while maintaining diagnostic performance by replacing non-enhanced image series with virtual non-enhanced series [7]. The clinical potential of spectral imaging for oncological patients is considered to be high [8]. Better demarcation of malignant lesions using dual-energy CT reconstructions has already been demonstrated for muscle metastases of malignant melanoma, head and neck masses or bone and liver metastases in multiple tumor entities [9,10,11]. Dual-layer CT can provide improved contrast between a tumor and the surrounding tissue using different techniques. Virtual monoenergetic image data (MonoE) can be produced with selective lower display energy than in conventional image data (CI), resulting in higher Hounsfield values of high atomic number materials [12,13,14]. This technique can facilitate the discrimination between the tumor and surrounding tissue by enhancing iodine contrast. At the same time, the iodine content in each image pixel can be quantified using material decomposition, which enables suppression of all other components and the display of only iodine on an absolute scale in iodine maps (IM) [15]. As demonstrated in phantom studies, quantification of iodine uptake has become sufficiently accurate to be used as a radiomic parameter [16,17]. As known from MRI studies, breast cancer presents hypervascularization, and the absence of enhancement in a lesion has a high negative predictive value [18,19]. Therefore, iodine quantification is a promising measure of enhancement in breast cancer.

The aim of this study was to investigate the diagnostic performance of breast lesion detection using novel dual-layer spectral CT. Two types of spectral image data, virtual monoenergetic image data at 40 keV display energy (MonoE40) and iodine maps (IM), were compared with conventional image data (CI), as acquired in a standard CT scan. On the one hand, a qualitative multi-reader assessment of breast cancer detection was performed for each image data set. On the other hand, a quantitative analysis of the breast masses was performed.

## 2. Materials and Methods

### 2.1. Patient Recruitment

For this prospective single-center, intra-individually controlled case-control study, institutional review board approval was granted and written informed consent was obtained from all patients. The inclusion criteria were histopathologically diagnosed unilateral breast cancer following ultrasound and mammography and a pending staging CT. The exclusion criteria were age < 18 years and previous therapy for breast cancer. 

### 2.2. Image Acquisition 

CT examinations of the thorax, abdomen, and pelvis were carried out 17.2 ± 4.1 days after biopsy. The examination protocol was a routine staging CT (IQon, Philips, Amsterdam, The Netherlands) at our institution according to our guidelines with no additional radiation exposure for study purposes. CT image data were acquired using a first-generation dual-layer spectral CT at 120 kVp. For CT image data acquisition parameters, see Table 1. 

The contrast agent Iohexol (ACCUPAQUE, GE HealthCare Technologies, Chicago, IL, USA) with an iodine content of 350 mg/mL was administered in a weight-adapted manner according to the following equation:

patient body weight (kg) × 1.43 mL/kg = volume of contrast agent (mL).

Image acquisition started with an arterial phase of the upper abdomen using the bolus-tracking technique with a region of interest (ROI) placed in the aorta at the level of the coeliac trunk. The acquisition threshold was 150 HU with a 13 s post-threshold delay. This arterial image series was for staging purposes only and was not used for the study. Following a delay of 60 s after bolus triggering, a venous phase image series of the thorax, abdomen, and pelvis was acquired. Axial reconstructions of virtual monoenergetic image data at 40 keV display energy (MonoE40), iodine maps (IM) and conventional CT image data (CI) were reconstructed using an iterative reconstruction algorithm (iDose level 6) with a slice thickness of 3.0 mm at an increment of 1.5 mm. 

### 2.3. Multi-Reader Analysis

**Figure 1:**
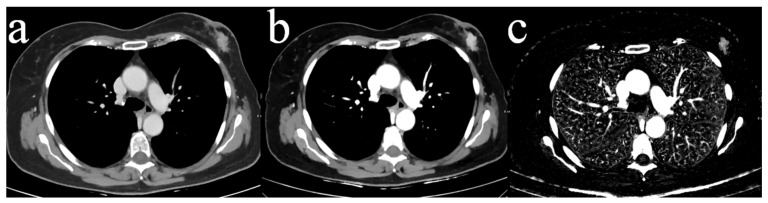
Reading example. Axial slice of the thorax showing a left-sided breast tumor at its maximum clip- and artifact-free diameter in the respective reading session. (**a**) Conventional image data (CI), (**b**) monoenergetic image data at 40 keV display energy (MonoE40), and (**c**) iodine maps (IM). Slice thickness 3 mm.

**Figure 2:**
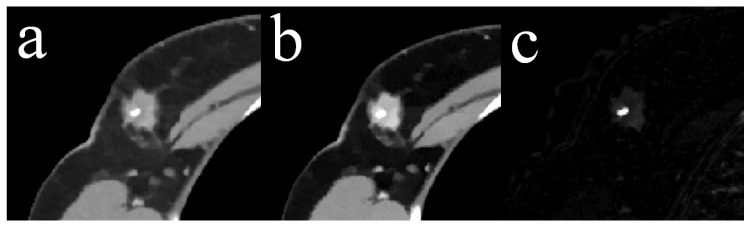
Example of biopsy artifacts. Axial slice of the thorax showing a right-sided breast tumor with a clip marker in the center. (**a**) Conventional image data (CI), (**b**) monoenergetic image data at 40 keV display energy (MonoE40), and (**c**) iodine maps (IM). Slice thickness 3 mm.

The locations and margins of 50 unilateral tumors in the 50 study patients were established by matching CT image data with the previous ultrasound/mammography examinations. Breast density scores (range *a*–*d*) were extracted from the reports of previous mammograms following ACR guidelines [20]. The four independent readers were experienced radiologists not involved in image selection and blinded to pathological and clinical data. Readers 1 (EW), 2 (AG), 3 (TM), and 4 (TW) had three years, seven years, ten years, and 15 years of experience in thoracic CT imaging, respectively. They underwent a training session using image data from ten patients not included in the study prior to the first reading session. The readers rated single slices of the thorax either showing regular breast tissue or a breast lesion in a randomized order. A total of 50 axial slices of both breasts were selected showing the unilateral tumor at its maximum clip- and artifact-free diameter as well as the contralateral breast (for an example, see Figure 1). The clip was omitted to avoid reader bias by evidence of prior biopsy. An example of biopsy artifacts omitted from the reads is shown in Figure 2. The 50 slices with regular breast tissue in each type of image data were defined by a distance of 6 slices from the tumor margin to the center of the breast and extracted accordingly. The readers were asked to note the presence or absence of a tumor in each breast and to rate the subjective diagnostic certainty of their decision for each breast on a five-point Likert scale (1 being the worst and 5 being the best score). There were three reading sessions: the first one for all CI acquired at 120 kVp, the second for all MonoE40, and the third for all IM image data. To minimize recall bias, there were four-week intervals between each reading session and the order of the image data was randomly changed. Sensitivity and specificity were calculated for each reader and each image type. 

### 2.4. Quantitative Measurements

Circular ROIs with a 5 mm diameter were placed in the area of the tumor with the highest CT number (HU) or iodine uptake, as well as in ipsilateral glandular tissue with no malignant features, in ipsilateral homogenous fat tissue of the breast, and the pectoralis major, all in the same slice. The contrast-to-noise ratio was calculated by subtracting the CT numbers measured in background tissue (glandular tissue) from the CT numbers measured in tumor tissue and dividing the difference by the standard deviation of the background tissue using the following formula:

$$CNR=\frac{HU\left(tumor\right)-HU\left(background\right)}{SD\left(background\right)}.$$

The signal-to-noise ratio was calculated as follows:

$$SNR=\frac{HU\left(tumor\right)}{SD\left(background\right)}.$$

For calculations based on iodine maps, the iodine content was used instead of the CT numbers. Iodine maps are displayed as red-green-blue color model (RGB) image data with a different scale (in mg/mL) and can therefore only be compared to the CT number measurements of the CI and MonoE data sets in a qualitative manner. 

### 2.5. Statistics

A paired *t*-test with Bonferroni correction for multiple comparisons was used for the comparison of two groups with parametric data, ANOVA for three groups, and a Mann–Whitney U test for non-parametric data. Inter-reader reliability was calculated by employing the Fleiss’ kappa test. κ values were interpreted in keeping with Landis and Koch [21] (0.21–0.40, fair agreement; 0.41–0.60, moderate agreement; 0.61–0.80, good agreement; 0.81–1.00, excellent agreement). Correlation of the non-metric data was evaluated using Spearman coefficient testing. The software used for statistical analysis was SPSS 25 (IBM, Armonk, NY, USA). A *p*-value of <0.05 was assumed to be statistically significant.

## 3. Results

A total of 50 female patients attended their examinations between October 2019 and January 2021. The median patient age was 51 years (ranging from 28 to 83 years). 

### 3.1. Multi-Reader Analysis

The sensitivity and specificity for breast cancer detection are given in Table 2 for each reader and image type.

**Figure 3:**
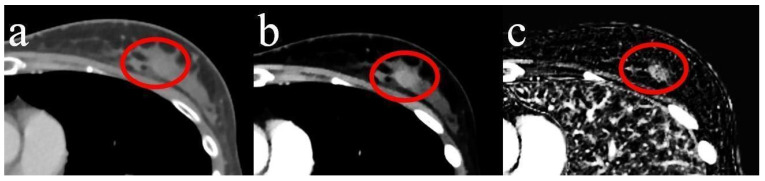
Breast cancer (circled in red) in the left breast within dense glandular tissue. (**a**) In conventional image data (CI), (**b**) in monoenergetic image data at 40 keV display energy (MonoE40), and (**c**) in iodine maps (IM). Slice thickness 3 mm.

The overall scores in sensitivity and specificity for CI were 0.90 and 0.92. MonoE40 yielded a sensitivity of 0.96 and specificity of 0.94. IM had the highest overall scores with 0.97 and 0.95. All of the differences in sensitivity and specificity between CI and MonoE40 or IM were statistically significant (*p* < 0.001). For an example of breast cancer that was missed by all of the readers in CI and MonoE40 but correctly detected by all four readers in IM, see Figure 3.

The Fleiss’ kappa inter-rater reliability was 0.80 for MonoE40, 0.78 for IM, and 0.74 for CI. The overall agreement was 0.91.

In patients with the highest breast density scores (ACR *d*), sensitivity was 0.59 for CI, 0.85 for MonoE40, and 1.00 for IM. Breast density as scored on a scale from *a* to *d* from lowest to highest was extracted from mammography reports. A score of *a* was found in 16 cases, *b* in 96 cases, *c* in 60 cases, and *d* in 26 cases.

**Figure 4:**
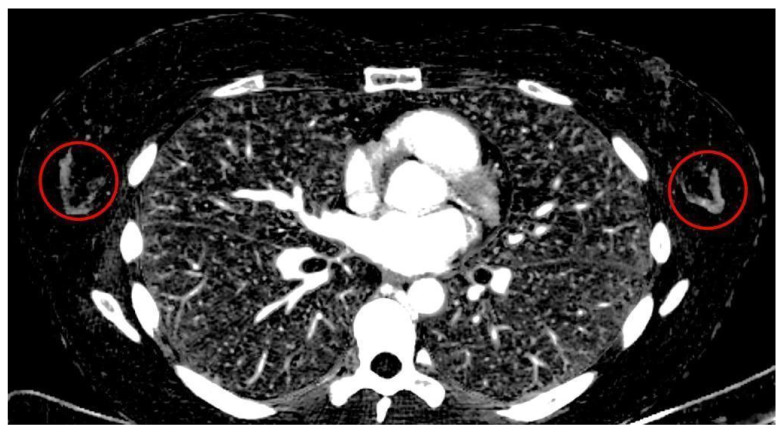
Symmetric background enhancement (circled in red) in an iodine map of a 35-year-old patient. Slice thickness 3 mm.

**Figure 5:**
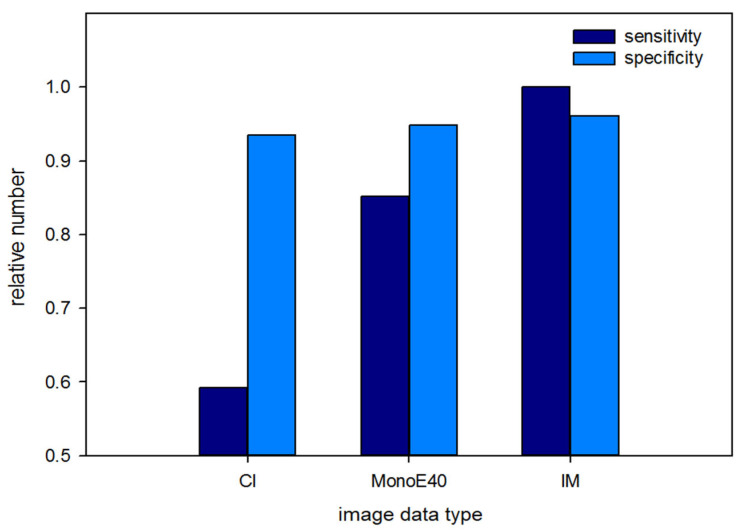
Overall sensitivity and specificity of different types of image data (conventional ‘CI’, monoenergetic at 40 keV display energy ‘MonoE40’, and iodine maps ‘IM’) in patients with high breast density (ACR *d*).

Among all readers there were a total of 21 false negatives/48 false positives in CI, compared to 9 false negatives/37 false positives in MonoE40 and 7 false negatives/28 false positives in IM. Therefore, the number of false negative assessments was 57% lower in MonoE40 and 67% lower in IM than in CI. The number of false positives was 23% lower in MonoE40 and 42% lower in IM than in CI. Among all readers, there was not a single case of false negative evaluation in patients with a breast density score of *d* in IM. For an example of high background enhancement in a 35-year-old patient in IM, see Figure 4. For overall sensitivity and specificity scores in patients with the highest breast density scores (ACR *d*), see Figure 5.

### 3.2. Subjective Diagnostic Certainty

For an overview of diagnostic certainty scores by reader and type of image data, see Table 3.

The overall subjective diagnostic certainty was highest in IM. The mean overall subjective diagnostic certainty was 3.5 ± 0.5 in CI, 4.1 ± 0.1 in MonoE40 and 4.5 ± 0.4 in IM (*p* < 0.001). For the more experienced readers, i.e., those with seven or more years of experience, subjective diagnostic certainty in MonoE40 was significantly higher than in CI (*p* < 0.001). For the most experienced readers, i.e., those with ten or more years of experience, subjective diagnostic certainty in IM was also significantly higher than in MonoE40 (*p* < 0.001).

### 3.3. Correlation Analysis

No significant correlation was found between breast density and correct lesion detection for any of the three types of image data with a Spearman coefficient of −0.07 (*p* = 0.06) for CI, −0.05 (*p* = 0.20) for MonoE40, and 0.04 (*p* = 0.29) for IM.

A weak but significant positive correlation between subjective diagnostic certainty and correctness of evaluation was found in all three types of image data with 0.23 for CI, 0.24 for MonoE40, and 0.20 for IM, respectively (*p* < 0.001).

Negative correlation of breast density and subjective diagnostic certainty was weak but significant for CI and MonoE40 with −0.28 and −0.23, respectively (*p* < 0.001). There was no correlation between breast density and subjective diagnostic certainty for IM at a significant level (−0.07, *p* = 0.04).

To check for recall bias, correlation between evaluations of the three reading sessions was calculated. The overall correlation of evaluations between the first and second reading session was 0.40 (*p* < 0.001) and 0.22 (*p* < 0.001) between the second and the third reading session.

### 3.4. Quantitative Assessment

The CNR of tumor vs. glandular tissue was significantly higher in MonoE40 than in CI image data with 36.8 ± 21.4 versus 23.7 ± 19.6 (*p* < 0.01). The CNR for IM was 11.3 ± 6.5. Likewise, the SNR was significantly higher in MonoE40 than in CI image data (*p* < 0.01) (Table 4).

Values for iodine content in the breast from fat to tumor ranged between 0 mg/mL and 6 mg/mL. Iodine uptake was significantly higher in tumor tissue than in regular glandular tissue with 1.9 ± 0.6 mg/mL vs. 0.1 ± 0.3 mg/mL (*p* < 0.0001). The average iodine uptake of fat tissue was 0.0 ± 0.1 mg/mL and did not differ significantly from the iodine uptake of regular glandular tissue (*p* = 0.94).

## 4. Discussion

This study demonstrated that spectral CT reconstructions including IM and MonoE40 are superior to CI regarding breast cancer detection with the best overall results for sensitivity, specificity and subjective diagnostic certainty for IM. The number of false evaluations of breast CT regarding the presence or absence of malignant tumors could be reduced significantly using MonoE40 or IM instead of CI. Both MonoE40 and IM yielded significantly better overall sensitivity and specificity than CI for breast cancer detection. This is consistent with the results on spectral imaging in other tumor entities [9,10,11]. MonoE40 and IM outperformed CI, especially in patients with high breast density. This can be explained by the suppression of normal glandular tissue in iodine maps, which has no relevant iodine uptake. Because the glandular tissue is almost completely obscured in iodine maps, there was no significant influence of breast density on detection rates when using this type of image data. We know breast tissue density is an important factor for tumor detection in screening; a recent study by Weigel et al. [22] found only 50% sensitivity for mammography in the population with the highest density score (ACR *d*).

Subjective diagnostic certainty was significantly higher in IM than in the other image data. With spectral image data, inter-rater reliability could also be improved, potentially facilitating consensus. Both higher subjective diagnostic certainty and inter-rater reliability can be explained by the suppression of normal glandular tissue resulting in less ambiguous image data.

In all three types of image data, there was only a weak but significant positive correlation between subjective diagnostic certainty and correctness of assessment regarding tumor presence. Thus, subjective diagnostic certainty as an indicator of correctness should not be overestimated when assessing breast tissue using CT. A weak but significant negative correlation between breast density and subjective diagnostic certainty could only be found in MonoE40 and CI. Breast density had no effect on subjective diagnostic certainty in IM, as non-attenuating breast tissue is suppressed in this imaging technique.

It should be noted that in rare cases of elevated background parenchymal enhancement of glandular tissue, MonoE40 image data have a lower CNR than CI and IM and yield a low diagnostic certainty. In these easily recognizable cases, spectral imaging does not provide diagnostic advantages. The assessment of patients with high background parenchymal enhancement is a well-known issue in breast MRI and recent data show an increased risk of breast cancer in this population [23].

It has already been established that CNR/SNR is higher in MonoE40 than in CI [24,25,26]. In the retrospective study by Okada et al., two readers evaluated the overall image quality and visibility of breast tumors in 42 breast cancer patients for a range of different energies (excluding IM) [24]. The readers were not blinded to the patients’ diagnosis. Readers rated tumor visibility significantly higher for MonoE40 and MonoE50 than for the CI equivalent. In similar retrospective study designs with 28 and 29 patients, respectively, Inoue et al. and Metin et al. also found better conspicuity of breast cancer in MonoE40 than in CI [25,26].

The iodine content of malignant tumors was significantly lower in the present study than in a comparable one with 31 patients, which can be explained by the higher iodine dose administered to the study patients by Volterrani et al. [27]. This highlights the importance of standardizing contrast agent dosage and application protocols for iodine quantification when using quantitative assessments. This is especially critical when using thresholds for distinguishing infiltrating carcinomas from other lesions, as Volterrani et al. suggest.

Buus et al. found a higher diagnostic performance of spectral CT imaging with IM and monoenergetic image data combined compared to CI in detecting breast cancer metastases in a prospective study with 182 patients [28]. Together with our study results, this makes spectral imaging especially attractive for follow-up examinations in breast cancer. Our results imply that contralateral secondary breast cancer and possibly also local recurrence might be detected more reliably with spectral CT imaging than with conventional CT, which would have to be verified in further studies.

Buus et al. found a higher per-lesion diagnostic performance for spectral CT than for whole-body MRI. Although the status of MRI in imaging the primary tumor is not challenged by our results, spectral imaging may take on special importance in patients with MRI contraindications. Although CT contrast agent is known to have a higher rate of side effects than its MRI counterpart, it is also worth noting that CT is more cost-efficient and, therefore, much more readily available. A disadvantage of breast MRI brought forward by Carbonaro et al. [29] is the prone patient positioning. MRI examination of subjects with breast lesions in prone and supine positioning showed displacements of lesions between 3 cm and 6 cm along different spatial axes. The standard positioning of breast MRI remains prone, whereas the standard positioning for breast sonography, breast surgery, and breast cancer staging in CT is supine. Thus, MRI positioning can produce distortions between cross-sectional imaging and ultrasound or intraoperative findings to which CT is less susceptible. Therefore, improved visualization of the primary tumor in CT could be useful for surgical planning, especially in patients with poorly delineated tumors in ultrasound and mammography. In this respect, future studies could focus on the comparison of tumor size in spectral CT and intraoperatively. Due to the high radiation exposure and the available as well as cost-efficient alternatives, CT will not be used as a screening method for breast masses. Spectral CT, however, has the potential to make optimal use of already acquired images beyond answering the clinical question.

Even though the benefits of spectral analysis in CT imaging of different diseases have already been demonstrated in numerous studies, these have so far only covered a small fraction of the possible applications. Spectral data can be acquired using modern dual-layer CT technology with no additional radiation exposure and this opens up a new dimension of image analysis by allowing qualitative parameters other than X-ray density to be analyzed. It is our responsibility to extract as much medically useful information as possible from an examination, especially when radiation exposure is involved. This study serves as a contribution to demonstrating the benefits of spectral data analysis for advancing future diagnostic applications.

### 4.1. Limitations

Although recall bias did not appear to be an issue of this study design as there was only a weak or moderate correlation of evaluations between consecutive reads, this study has several limitations.

First, the sample size was relatively small. This was due to the design as an initial, prospective, hypothesis-generating study.

Second, no benign breast lesions like fibroadenomas were among the study image data. Thus, only a distinction between malignant breast tumors and normal breast tissue could be made. Therefore, the found quantitative iodine levels, especially in tumor tissue, can only provide orientating values for future clinical studies. It goes without saying that a control group with benign breast lesions cannot be prospectively studied using CT for study purposes following ethical standards because of the radiation exposure involved.

Third, the median age in the study population was significantly lower than the median worldwide age at diagnosis [30]. This is likely to have led to higher average breast density in the study population which could cause an overestimation of the benefits of spectral imaging but also an underestimation of sensitivity and specificity in the target population. No data were collected on the menstrual cycle of the patients. A cycle dependence of the results in premenopausal patients cannot be excluded.

Fourth, we decided to present the readers with only single slices and not a 3D-stack of the patients’ image data due to the presence of metal clips marking the tumor or suspicious lymph nodes, or other traces of recent biopsy. The presence of biopsy artifacts would have undoubtedly caused strong reader bias and would most likely have influenced the readers’ decision because they would have suggested suspected malignancy in the patient’s medical history. As all clip markers in the study patients originated from tumor biopsies, the specificity would have been systematically increased in all modalities. The tumor incidence in the study was significantly higher than in the normal population, which means that the absolute values of sensitivity and specificity cannot be applied to the latter. However, because both sensitivity and specificity were higher in MonoE40 and IM, the superiority of spectral imaging can be assumed even at a lower incidence of breast cancer.

Fifth, the benefit from facilitated decision making due to higher subjective diagnostic certainty in IM compared to CI could be outweighed by the extra time required for the iodine map review. Reading time was not recorded to account for these factors.

### 4.2. Conclusions

With spectral CT, breast cancer can be detected more reliably with higher sensitivity and specificity. Subjective diagnostic certainty and inter-reader reliability can also be increased. Iodine maps are especially helpful in patients with dense glandular tissue. Spectral imaging may thus facilitate the incidental detection of breast cancer on CT performed for other reasons in the future. The results highlight the value of spectral image data by extracting valuable information from already available images.

## Figures and Tables

**Table 1:** CT image data acquisition parameters.

General	
Tube potential (kVp)	120
Tube current-time product (mAs)	74
Collimation (number of slices × slice thickness in mm)	64 × 0.625
Gantry rotation time (s)	0.5
Pitch	0.798
CTDIvol (mGy)	7.5
Post-threshold delay (s)	60
Image reconstruction	
Slice thickness/increment (mm)	3.0/1.5
Window width (HU)	400
Window center (HU)	40
Field of View (mm)	350
Matrix	512 × 512

**Table 2:** Sensitivity and specificity for breast cancer detection. Sensitivity and specificity for the detection of breast cancer for each reader and image data type (conventional ‘CI’, monoenergetic at 40 keV display energy ‘MonoE40’, and iodine maps ‘IM’). Best results of each reader in bold.

	CI		MonoE40		IM	
	Sensitivity	Specificity	Sensitivity	Specificity	Sensitivity	Specificity
Overall	0.90	0.92	0.96	0.94	0.97	0.95
Reader 1	0.92	0.85	**0.98**	0.82	**0.98**	**1.00**
Reader 2	0.94	0.90	0.94	**0.97**	**1.00**	0.85
Reader 3	0.84	0.95	0.96	**0.98**	**1.00**	0.96
Reader 4	0.88	0.98	**0.94**	0.98	0.88	**1.00**

**Table 3:** Overview of diagnostic certainty scores. Mean ± standard deviation of subjective diagnostic certainty (for each reader and type of image data (conventional ‘CI’, monoenergetic at 40 keV display energy ‘MonoE40’, and iodine maps ‘IM’). Best results of each reader in bold.

	CI	MonoE40	IM
Reader 1	4.2 ± 0.8	4.0 ± 0.8	**4.4 ± 0.8**
Reader 2	3.4 ± 1.4	**4.1 ± 1.1**	4.0 ± 1.0
Reader 3	3.6 ± 1.4	4.1 ± 1.2	**4.8 ± 0.6**
Reader 4	3.0 ± 1.3	4.2 ± 0.8	**4.8 ± 0.6**

**Table 4:** CNR and SNR values. Mean ± standard deviation of contrast-to-noise ratio (CNR) and signal-to-noise ratio (SNR) of tumor tissue for each type of image data (conventional ‘CI’, monoenergetic at 40 keV display energy ‘MonoE40’, and iodine maps ‘IM’).

	CI	MonoE40	IM
CNR	23.7 ± 19.6	36.8 ± 21.4	11.3 ± 6.5
SNR	30.7 ± 16.4	41.2 ± 22.2	12.0 ± 6.5

## Data Availability

The authors will provide access to anonymized data upon reasonable request.

## Abbreviations

ACR	American College of Radiology
ANOVA	Analysis of Variance
CI	conventional CT image data
CNR	Contrast-to-noise ratio
CT	Computed tomography
HU	CT value in Hounsfield units
IM	iodine maps
MonoE40	monoenergetic image data at 40 keV display energy
MRI	magnetic resonance imaging
SNR	Signal-to-noise ratio
RGB	red, green, and blue additive color model
ROI	region of interest
